# Gut microbial dysbiosis is associated with allergen-specific IgE responses in young children with airway allergies

**DOI:** 10.1016/j.waojou.2019.100021

**Published:** 2019-03-25

**Authors:** Chih-Yung Chiu, Yi-Ling Chan, Ming-Han Tsai, Chia-Jung Wang, Meng-Han Chiang, Chun-Che Chiu

**Affiliations:** a Department of Pediatrics, Chang Gung Memorial Hospital at Keelung, Chang Gung University, Taoyuan, Taiwan; b Division of Pediatric Pulmonology, Department of Pediatrics, Chang Gung Memorial Hospital at Linkou, Chang Gung University, Taoyuan, Taiwan; c Department of Emergency Medicine, Chang Gung Memorial Hospital at Linkou, Taoyuan, Taiwan; d Clinical Informatics and Medical Statistics Research Center, Chang Gung University, Taoyuan, Taiwan; e Clinical Metabolomics Core Laboratory, Chang Gung Memorial Hospital at Linkou, College of Medicine, Chang Gung University, Taoyuan, Taiwan

**Keywords:** Asthma, *Clostridium* spp., *Dorea* spp., Fecal IgE, Gut microbiota, IgE, immunoglobulin E, OTUs, Operational taxonomic Units, QIIME, Quantitative Insights into Microbial Ecology

## Abstract

**Background:**

There is increasing evidence linking alterations of the gut microbial composition during early infancy to the development of atopic diseases and asthma. However, few studies have addressed the association of dysbiotic gut microbiota with allergic reactions through evaluation of feces in young children with allergic airway diseases.

**Methods:**

We sought to evaluate relationships among gut microbiota, total fecal immunoglobulin E (IgE) levels, serum allergic sensitization, and their relevance to childhood allergic rhinitis and asthma. Microbial composition and diversity were analyzed with Illumina-based 16S rRNA gene sequencing of 89 stool samples collected from children with asthma (n = 35) and allergic rhinitis (n = 28), and from healthy controls (n = 26). Data analysis was performed using Quantitative Insights into Microbial Ecology (QIIME) software.

**Results:**

A significantly lower abundance of organisms of the phylum Firmicutes were found in children with asthma and allergic rhinitis than in the healthy controls. Relatively lower Chao1 and Shannon indices were also found in children with allergic airway diseases but without any significant difference. Total fecal IgE levels in early childhood were strongly correlated with serum *D. pteronyssinus*- and *D. farinae*-specific IgE but not with food-specific IgE levels. In comparison with healthy controls, the genus *Dorea* was less abundant and negatively correlated with total fecal IgE levels in children with rhinitis, whereas the genus *Clostridium* was abundant and positively correlated with fecal IgE levels in children with asthma.

**Conclusions:**

An interaction between particular subsets of gut microbial dysbiosis and IgE-mediated responses to allergens may contribute to the susceptibility to allergic rhinitis and asthma in early childhood.

## Background

Allergic airway diseases, such as rhinitis and asthma, are common chronic inflammatory disorders which are a major health issue in children.^1^ Several socio-demographic and individual factors are significantly associated with atopic symptoms and diseases.^2^, ^3^, ^4^ Allergen sensitization is known to be an important factor in childhood atopic diseases.^5^ Hosts and their microbiomes have evolved symbiotic relationships and this relationship has been implicated in the regulation of mucosal immunity and inflammation.^6^ An interaction between dysbiotic states of microbiota and allergic reactions in response to allergen exposure plays an important role in allergic airway diseases.^7^

A recent longitudinal analysis has demonstrated a link between the gut microbe-environment interactions and the development of childhood asthma.^8^ Gut microbiota co-evolves with the infants’ immune system, and several studies have found that altered microbial diversity in early infancy precedes the development of allergic rhinitis and asthma at school age.^9^, ^10^, ^11^ However, the prevalence of rhinitis and asthma increases exponentially after infancy.^2^, ^12^ Despite evidence linking early dysbiosis of the gut microbiome to allergies,^13^ few studies have addressed the impact of gut microbiota on allergen sensitization and atopic diseases in early childhood.

Serum immunoglobulin E (IgE) is an antibody produced in response to allergy and this allergen-specific IgE is integral to the pathogenesis of allergic disorders. IgE is also produced locally in the gut as a result of stimulation by food allergens and serves as an indicator of food sensitization.^14^ Furthermore, an altered pattern of gut microbiota in the early stages of life is implicated in the risk of IgE-mediated food allergy in children.^15^ Clinically, mite-specific IgE appears to be significant in childhood rhinitis and asthma.^2^ However, it is still not very clear how the fecal and serum allergen-specific IgE levels and gut microbiota for allergic airway diseases are linked during early childhood.

A detailed understanding of the interactions between the external allergen exposure, gut microbial diversity, and host immunocompetence will most likely provide valuable clinical insights into therapeutic strategies for gut microbiota modulation in allergic airway diseases. The aim of this study was to examine the gut microbial profiles in patients with childhood rhinitis, asthma along with the healthy controls. The relationship between the composition and diversity of the gut bacteria and atopic indices such as fecal and serum allergen-specific IgE levels were assessed and their relevance to allergic rhinitis and asthma was also examined.

## Methods

### Study population and data collection

A cross-sectional controlled study was designed to investigate the gut microbiota profiles in children with asthma, allergic rhinitis and healthy controls. Children aged between 4 and 7 years old who diagnosed with asthma alone or rhinitis alone, and healthy controls were consecutively recruited from November 1, 2015 to October 31, 2017 for this study. The phenotypes of atopic diseases were physician-diagnosed and evaluated by the same pediatric pulmonologist at the outpatient clinics. Asthma was diagnosed as having the occurrence of non-infectious recurrent wheeze in the last 12 months, or current use of asthma medication, based on the guidelines of the Global Initiative for Asthma.^16^ Allergic rhinitis was diagnosed as having symptoms such as sneezing, nasal congestion, itching, and rhinorrhea in the last 12 months.^17^ Healthy controls without a history of asthma or other atopic conditions or infections were enrolled and paired. Children who present with a combination of asthma and rhinitis when diagnosed were excluded. Detailed information on potential confounding variables for atopic diseases including sex, maternal atopy, passive smoking, and household income were recorded and analyzed. This study was approved by the Ethics Committee of Chang Gung Memory Hospital (No. 104-3757B). Written informed consent was obtained from the parents or guardians of all the study subjects.

### Sample collection and storage

Fresh stools were collected in clean specimen bottles by parents from each child with instructions on proper method of collection. Stool samples were frozen immediately, and carefully transported to our laboratory where they were stored at −80 °C until further use. None of the subjects had received antibiotics for at least four weeks prior to the sampling.

### Measurement of serum and fecal IgE levels

Total serum and allergen-specific IgE levels were examined as described previously.^18^ Allergen-specific IgE levels were determined using a commercial assay for IgE (ImmunoCAP Phadiatop Infant; Phadia) for a mix of two most common food allergens (egg white and cow's milk) and two most common aeroallergens causing sensitization in more than 95% of children in Taiwan (*Dermatophagoides pteronyssinus* and *Dermatophagoides farina*).^19^ For total fecal IgE levels, 1.5 g of lyophilized stool samples were diluted with 3 mL deionized water and homogenized for 1 min. After centrifugation at 10,000 rpm for 30 min at 4 °C (Eppendorf centrifuge 5810R), the supernatants were collected and stored at −80 °C until further use. Total fecal levels of IgE were measured using Immunoglobulin E ELISA Kit (Immundiagnostik AG, Bensheim, Germany) according to the manufacturer's instructions.

### DNA extraction, 16S rRNA gene amplification, and sequencing

Bacterial DNA was extracted from the same amount of feces (0.5 g) using a FastDNA Spin Kit for Feces (MP Biomedical, Solon, OH, USA) following the manufacturer's instructions. DNA was extracted with 70 μL of TE buffer and the purity was quantified by measuring the absorbance at 260 and 280 nm with a spectrophotometer (Nanodrop 1000; Thermo Scientific, Waltham, MA, USA). All samples had an A260-to-A280 absorbance ratio between 1.8 and 2.1. Polymerase chain reaction (PCR) was used to amplify the variable region V3—V4 of the gene that encodes for 16S rRNA in bacteria by using bacteria/archaeal primer 515F/806R with the barcodes.^20^ Gel electrophoresis of PCR products on 2% agarose gels was performed for quality control. Samples with one clear band between 400 and 450 bp were selected for further experiments. Amplicons were purified using the GeneJET Gel Extraction Kit (Thermo Scientific) and then quantified using a Qubit dsDNA HS Assay Kit (Qubit) on a Qubit 4.0 Fluorometer (Qubit). Sequencing libraries were generated using NEB Next^®^ Ultra™ DNA Library Prep Kit for Illumina (NEB) following manufacturer's recommendations. Purified libraries were quantified, normalized, pooled, and applied for cluster generation and sequencing on an Illumina HiSeq 2500 platform (Illumina, Inc., San Diego, CA, USA) according to the manufacturer's instructions. The sequence data and mapping file for all the samples included in this study have been deposited in Figshare (https://figshare.com/s/6b545fe1555c345d0f9e).

### Sequence processing and data analysis

Amplicon was performed by using paired-end 250 bp reads, and then assembled and pretreated to obtain Clean Tags using FLASH.^21^ Reads less than 100 nucleotides and chimeric sequences were detected and removed to obtain the effective tags finally using UCHIME algorithm.^22^ Data analysis was performed using the software ‘‘Quantitative Insights into Microbial Ecology’’ (QIIME).^23^ Assembled sequences were clustered into Operational taxonomic Units (OTUs) using Uparse software at 97% sequence identity,^24^ and taxonomy classification was assigned based on full-length 16S rRNA gene database, Greengenes.^25^

Bacterial community profiles were analyzed and rarefaction curves based on the number of species were generated for each sample from randomized OTU draws. Microbial community comparisons were made using the Bonferroni correction test which corrected the *P*-values for multiple comparisons in Unifrac.^26^ Abundance differences between groups were tested using the MetaStat method with multiple comparison adjustments.^27^ As described in our previous study,^7^ richness of each sample was calculated with the Chao1 index and diversity accounting for both relative abundance and evenness was evaluated with Shannon index. Beta diversity was calculated between groups at OTU genus level and Principal Coordinate Analysis (PCoA) plot in conjunction with weighted Unifrac and non-metric multidimensional scaling (NMDS) plot in two-dimensional taxon space based on Bray-Curtis similarities were produced to show clustering between groups.^26^, ^28^ Similarities analysis was conducted using the unweighted-pair group method with the arithmetic average (UPGMA) clustering algorithm. Rare OTUs were defined as less than 0.01% of the reads in a given sample, and were removed if more than 50% of all samples.^29^

### Statistical analysis

Comparisons of the baseline characteristics among the asthma patients, rhinitis patients, and the healthy controls were performed using univariable parametric and non-parametric tests such as analysis of variance (ANOVA), Kruskal-Wallis test, and chi-square test respectively. All continuous variables were analyzed using the Mann-Whitney test for comparison between two groups and the Kruskal-Wallis test for comparison between three groups. Pearson's or Spearman's correlation tests were used to determine the correlations of the relative abundance of the microorganisms with total fecal IgE levels and serum allergen specific IgE levels. Statistical analysis was performed by using the Statistical Package for the Social Sciences (SPSS Statistics for Windows Version 20.0; Armonk, NY, USA). All statistical hypothesis tests were two-tailed and a *P*-value < 0.05 was considered significant.

## Results

### Population characteristics

Eighty-nine subjects were enrolled in this study, including 35 children with asthma, 28 children with allergic rhinitis, and 26 healthy controls. The baseline characteristics of the children with asthma, rhinitis, and the healthy controls are shown in Table 1. The average age of the subjects was 5.7 years (range: 4.4–6.8 years) which was similar for all the groups. Atopic indices including total fecal IgE levels and total serum and *D. pteronyssinus*-specific IgE levels were significantly higher in children with asthma and rhinitis than in the healthy controls. There was no significant difference in other characteristics such as sex, maternal atopy, passive smoking, and household income between the three groups.

**Table 1:** Epidemiologic characteristics of the 89 children investigated in this study.

Characteristics	Controls	Asthma	Rhinitis	*P*-value
	(n = 26)	(n = 35)	(n = 28)	
Age (yr)	5.6 ± 0.8	5.5 ± 0.9	5.9 ± 0.9	0.245
Sex, male	14 (53.8%)	21 (60.0%)	17 (60.7%)	0.852
Maternal atopy	11 (42.3%)	20 (64.5%)	13 (46.4%)	0.195
Passive smoking	10 (38.5%)	14 (41.2%)	13 (46.4%)	0.832
Household income				0.379
Low, ≤ 500,000 NTD	10 (38.5%)	13 (37.1%)	10 (35.7%)	
Medium, 500,000–1,000,000 NTD	12 (46.2%)	16 (45.7%)	9 (32.1%)	
High, > 1,000,000 NTD	4 (23.5%)	5 (14.3%)	9 (32.1%)	
Allergen-specific IgE, kU/L				
*D. pteronyssinus*	6.1 ± 20.5	31.4 ± 34.5	32.5 ± 39.7	0.013
*D. farinae*	4.6 ± 17.3	22.1 ± 28.0	21.5 ± 31.2	0.051
Egg white	0.3 ± 0.7	0.7 ± 1.1	0.4 ± 0.8	0.364
Cow's milk	0.2 ± 0.4	0.5 ± 0.7	0.3 ± 0.4	0.250
Total serum IgE, kU/L	88.3 ± 142.6	429.0 ± 591.3	313.0 ± 476.1	0.003
Total fecal IgE, kU/L	3.6 ± 7.9	7.0 ± 6.0	15.2 ± 27.7	<0.001

Data shown are mean ± SD or number (%) of patients as appropriate. yr, year; NTD, New Taiwan Dollar; IgE, immunoglobulin E.

### Gut bacterial community composition and abundance

**Fig. 1:**
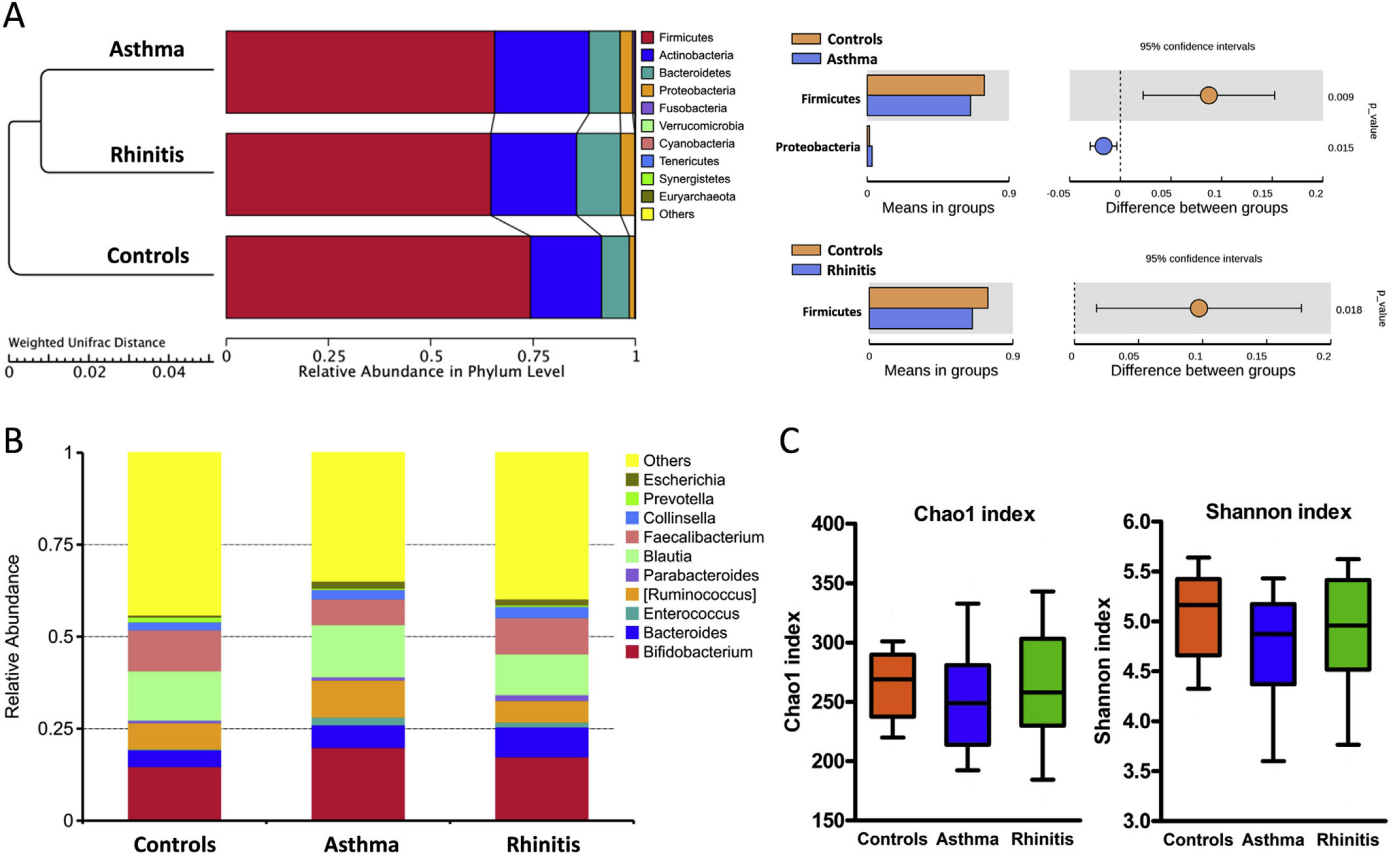
Fecal microbial composition and abundance at the phylum and genus level. (A) UPGMA clustering trees based on weighted Unifrac distance and Student's *t*-test bar plot of bacterial taxon phyla. (B) Bacterial composition and abundance in genus level. Each bar represents the top ten bacterial species ranked by the relative abundance in children with asthma, rhinitis and healthy controls. (C) Differences and comparisons of species richness and diversity among atopic diseases. Species richness calculated as the Chao1 index. Species diversity calculated as the Shannon index. The box-plot shows the median and the 10th, 25th, 75th and 90^th^ percentile.

The reads obtained from stool microbiota was more than 20,000 and a total of 150–250 OTUs were detected. Rarefaction curves showed that a plateau of species richness was achieved at 23,254 reads per samples (Additional file 1: Figure S1). Randomly, 23,000 reads were then used as the minimum sampling depth to capture diversity. The bacterial composition and abundance at the phylum and genus levels are shown in Fig. 1A and B. The taxonomic classification showed a high prevalence of members of the phylum Firmicutes (67.8% of the total number of sequences obtained) followed by those of the phyla Actinobacteria (20.7%), Bacteroidetes (8.4%), Proteobacteria (2.7%), and others. *Bifidobacterium* (17.6%), *Blautia* (13.0%), *Faecalibacterium* (9.1%), *Ruminococcus* (7.9%), and *Bacterioides* (6.3%) were the top five most predominant genera.

### Differential abundance analysis of the gut microorganisms for asthma and rhinitis

At phylum level, a significantly lower abundance of Firmicutes was found in children with asthma and rhinitis (*P* = 0.009 and *P* = 0.018, respectively), compared to the healthy controls. In contrast, the phylum Proteobacteria was predominant in children with asthma in comparison with healthy controls. Differences in the abundance of members belonging to different genera among the children with asthma, rhinitis, and the healthy controls are shown in Table 2. In children with asthma, the members of the genera *Roseburia*, *Faecalibacterium*, *SMB53*, and *Ruminococcus* was significantly less abundant as seen in case of the genus *Dorea* in children with rhinitis. However, the genus *Escherichia* of the phylum Proteobacteria and the genus *Clostridium* of the phylum Firmicutes were significantly predominant in children with asthma.

**Table 2:** Differences and comparisons of bacteria in phyla and genera among children with asthma, rhinitis and healthy controls.

Phylum/Genus	Controls (n = 26)	Asthma (n = 35)	Rhinitis (n = 28)	Asthma vs. Controls	Rhinitis vs. Controls
	mean ± SD (%)	mean ± SD (%)	mean ± SD (%)	FDR *P*-value	FDR *P*-value
Proteobacteria/*Escherichia*	0.59 ± 0.50	1.98 ± 2.43	1.68 ± 4.22	0.002	
Firmicutes/*Eubacterium*	0.05 ± 0.04	0.13 ± 0.12	0.06 ± 0.07	<0.001	
Firmicutes/*Roseburia*	5.71 ± 4.21	3.03 ± 3.22	3.74 ± 4.01	0.009	
Firmicutes/*Faecalibacterium*	11.17 ± 6.51	7.00 ± 6.33	9.79 ± 6.33	0.015	
Firmicutes/*Clostridium*	0.85 ± 0.64	1.22 ± 1.07	1.43 ± 1.63	0.029	
Firmicutes/*SMB53*	3.72 ± 3.47	2.10 ± 1.27	2.68 ± 2.04	0.030	
Firmicutes/*Ruminococcus*	3.48 ± 1.94	2.44 ± 1.95	4.20 ± 3.04	0.045	
Firmicutes/*Phascolarctobacterium*	0.05 ± 0.06	0.10 ± 0.14	0.08 ± 0.10	0.048	
Firmicutes/*Dialister*	0.42 ± 0.53	0.12 ± 0.18	0.13 ± 0.14	0.009	0.013
Firmicutes/*Dorea*	2.07 ± 1.32	2.27 ± 1.70	1.44 ± 0.77		0.040
Actinobacteria/*Adlercreutzia*	0.05 ± 0.06	0.07 ± 0.10	0.12 ± 0.14		0.027

Data shown are mean ± SD of relative abundance of bacteria. The percent of total numbers of sequences are shown for each split level. Only taxonomic classification with more than 100 sequences or 0.01% of total sequences, and statistically significant differences are shown. *P*-values are calculated by MetaStat method with FDR adjustment for multiple testing.

### Bacterial richness and diversity categorized by atopic diseases

Relatively lower Chao1 and Shannon indices were found in children with asthma and rhinitis than in the healthy controls, but these differences were not significant (Fig. 1C). Furthermore, bacterial richness and diversity were not considerably different in relation to risk variables for atopic diseases such as sex, maternal atopy, passive smoking, and household income. Beta diversity statistics using the Principal Coordinate Analysis (PCoA) and non-metric multidimensional scaling (NMDS) revealed no significant differences in the microbial communities cluster patterns with regard to allergic airway diseases (Additional file 2: Figure S2).

### Microorganisms associated with total fecal and serum allergen-specific IgE levels

**Fig. 2:**
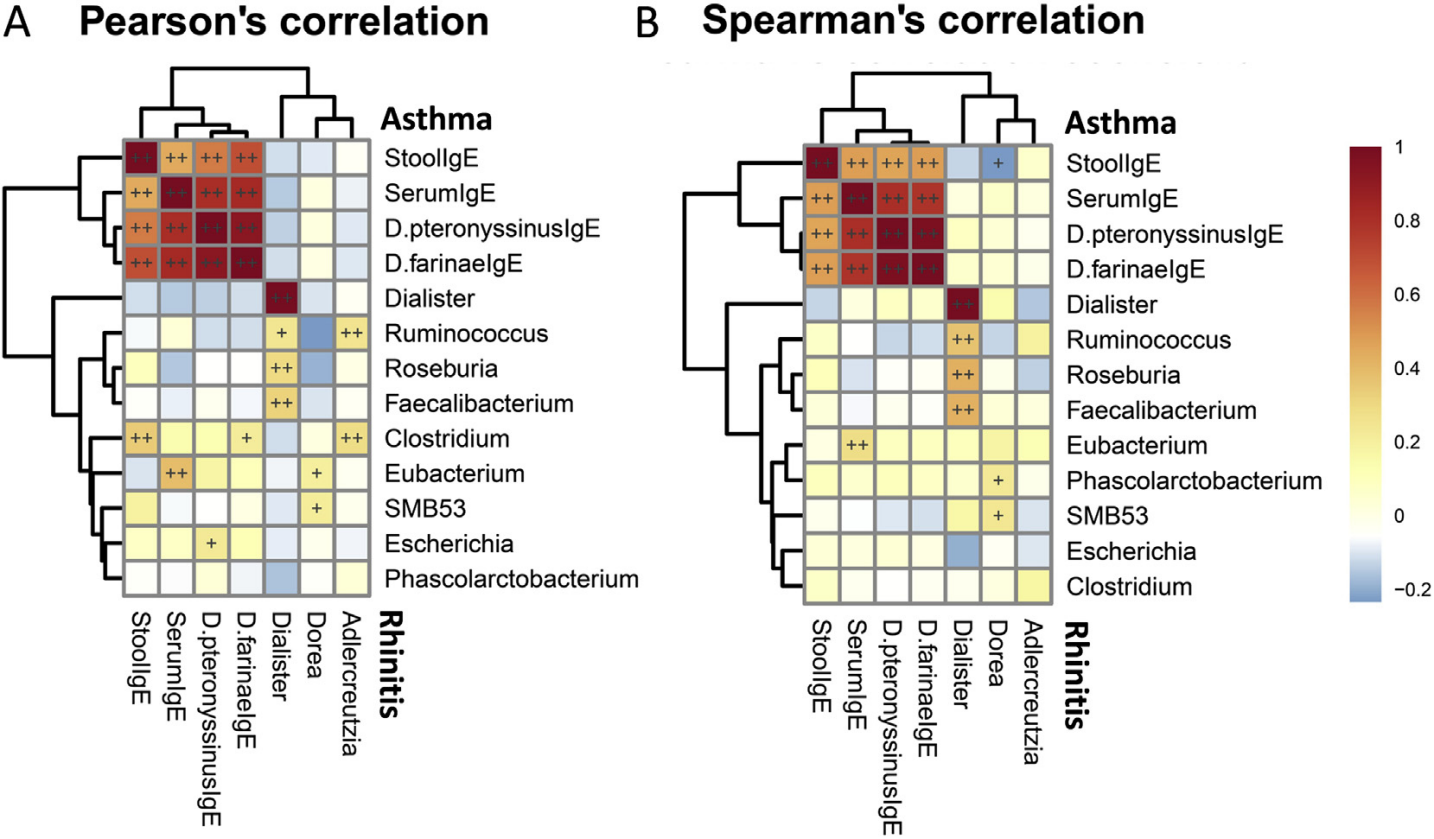
Heatmaps of correlations of microorganisms with total fecal and allergen-specific IgE levels for rhinitis and asthma. Correlations between significantly expressed genera of bacteria involved in rhinitis and asthma, and total fecal and serum allergen-specific IgE levels using Pearson's coefficient (A) and Spearman's rank coefficient (B). Color intensity represents the magnitude of correlation. Red color represents positive correlations; blue color represents negative correlations. + symbol means a *P*-value < 0.05; ++ symbol means a *P*-value < 0.01.

The total fecal and allergen-specific IgE levels were correlated and analyzed. Total fecal IgE levels were strongly correlated only with serum *D. pteronyssinus*- and *D. farinae*-specific IgE levels (Fig. 2). Microorganisms found to be involved in asthma and rhinitis were then correlated with fecal and serum allergen-specific IgE levels. *Clostridium* spp. and *Escherichia* spp. were significantly positively correlated with *D. farinae* and *D. pteronyssinus* IgE levels, respectively (Fig. 2A). Furthermore, a positive correlation was found between *Clostridium* spp. and total fecal IgE levels, whereas a negative correlation was found between *Dorea* spp. and fecal IgE levels (Fig. 2B).

## Discussion

Dysbiotic gut microbiomes in early infancy are at higher risk for atopy development later in life.^30^, ^31^ However, allergic airway diseases frequently develop in childhood accompanying with the changes in sensitization to aeroallergens. In this study, our comprehensive investigation has demonstrated a link between dysbiosis of particular subsets of the gut microbiota and IgE-mediated allergic responses for allergic rhinitis and asthma in early childhood.

Several studies have supported that altered gut microbiota is related to a higher prevalence of atopic disease and asthma.^32^, ^33^ The early diversity of gut microbiota could be critical for microbe-host interactions contributing to the immune system and atopic disease development. Recent studies have also found that reduced gut microbial diversity during infancy precedes the development of atopic diseases later in life.^9^, ^10^ However, our findings showing no association between gut bacterial diversity and allergic airway diseases in early childhood indicate that the deviations in the gut microbial development play an important role in the later development of atopic diseases.

The role of microbiota in health and food allergy has been established with reference to the human gut.^34^ Smaller proportions of Bacteroidetes and larger proportions of Firmicutes have been reported to be associated with food-allergic infants.^35^ However, sensitization to aeroallergens occurs after infancy, specially, in the development of rhinitis and asthma in early childhood.^2^ In this study, in contrast to previous studies conducted on infants, Firmicutes appeared to be lower in school children with allergic airway diseases. This conflicting finding indicates that the ecological succession of Firmicutes may take part in the allergic response changes to aeroallergens in childhood allergic rhinitis and asthma.

An allergic reaction occurs when the immune system overreacts to an allergen by producing specific IgE antibodies.^36^ Fecal IgE levels represent food allergen exposure and food sensitization during infancy.^14^ However, in early childhood, the IgE concentrations in feces were correlated with corresponding serum total IgE levels and specific IgE levels to mites, but not to allergens in egg white or cow's milk. This may be due to the fact that food sensitization decreases in children after infancy which in turn, reduces statistical efficiency. Our findings indicate that fecal IgE levels can be a promising indicator of allergic conditions in response to allergen exposure.

Innate response to microbial components has been reported to modulate the immunity of children with allergic diseases by the aberrant immune maturation of adaptive T-cells response to allergens.^37^ A reduced relative abundance of *Ruminococcus* spp. in the gut is associated with Toll-like receptors induced inflammatory responses and subsequent development of eczema in infancy.^38^ In this study, in addition to *Ruminococcus* spp., members of the phylum Firmicutes including the genera *Faecalibacterium*, *Roseburia*, *SMB53*, and *Dialister* were observed to be depleted in the gut of patients with childhood asthma. However, they did not correlate with fecal IgE or allergen-specific IgE levels. Thus, our results indicate that depletion of certain bacteria such as the Firmicutes may modulate the immune function of T-cells by presenting antigens contributing to asthma rather than eliciting adaptive immune responses by modulating B-cell antibody production.

In contrast, the genus *Dorea* of the phylum Firmicutes has been reported to be inversely associated with both food sensitization and food allergy in infancy.^39^ Similarly, in early childhood, *Dorea* spp. were inversely correlated with *D. pteronyssinus*-specific IgE levels and appeared to be more specific to allergic rhinitis as shown in this study. Because of the persistent association of genus *Dorea* with allergy, an alteration in the composition of the *Dorea* spp. may lead to prevention or treatment of allergic rhinitis by altering the development of allergic sensitization to house dust mite.

*Clostridium* species from the phylum Firmicutes have been shown to strongly influence host immunity, and its colonization in very early life is associated with an increased risk of allergy.^40^ In this study, the genus *Clostridium* was consistently predominant in early childhood and was associated with asthma. Furthermore, a strong correlation was observed between *Clostridium* spp. and fecal IgE and serum *D. pteronyssinus*-specific IgE expression. In addition, *Escherichia* spp. from the phylum Proteobacteria also appeared to be correlated with mite sensitization related to asthma. These observations elucidate a potential role of certain bacteria in atopic diseases through suppression of immunological tolerance to allergens.^41^

The major limitation of this study is the relatively small sample size that may understate potential differences. In addition, the difficulty in identifying all bacterial species in the gut flora limits the ability to fully resolve the identity of the taxa that may be important for the development of allergic airway diseases. However, an age-matched comparison group of healthy children without any atopy-related symptoms eliminates the dissimilarities in microbial compositions amongst a wide range of age groups. Repeated and careful characterization of the developing allergic phenotype at outpatient clinics also reduces the risk of misclassification. Most importantly, a direct investigation of the link between gut microbiota and IgE production in the feces of children with allergic diseases could represent the impacts of gut microbial dysbiosis on allergy, which makes our results valid and potentially important.

## Conclusions

This study provides evidence that alterations in the composition of the gut microbiota have an association with allergic reactions to antigens contributing to childhood allergic airway diseases. In early childhood, total fecal IgE levels appear to be specifically correlated with house dust mite-specific IgE levels, indicating that fecal IgE levels represent markers of allergic response to aeroallergens. A significant correlation of fecal IgE levels with *Dorea* spp. and *Clostridium* spp. related to allergic rhinitis and asthma respectively suggests that modulation of particular subsets of gut microbial dysbiosis and subsequently, the allergen responses could potentially contribute to the susceptibility to allergic airway diseases. However, further work is required for the identification of specific species and functional studies as well as to understand the strength and mechanism of these associations.

## Availability of data and materials

The datasets used and/or analyzed during the current study are available from the corresponding author on reasonable request.

## Ethics approval and consent to participate

This study was approved by the Ethics Committee of Chang Gung Memory Hospital (No. 104-3757B). Written informed consent was obtained from the parents or guardians of all the study subjects.

## Consent for publication

All authors have approved the manuscript for submission.

## Funding

This study was financially supported by research grants of CMRPG2G0651 and CMRPG2E0301 from the Chang Gung Memorial Hospital, Taiwan, and MOST 107-2314-B-182A-107 of the Ministry of Science and Technology in Taiwan.

## Competing interests

The authors declare that they have no competing interests.
