# Supplementary material for: Gut microbial dysbiosis is associated with allergen-specific IgE responses in young children with airway allergies
Source: World Allergy Organ J. 2019 Mar 25;12(3):100021. doi: 10.1016/j.waojou.2019.100021 (PMC6439417; doi:10.1016/j.waojou.2019.100021)
Supplement: Multimedia component 1 [file mmc1.docx]

**Additional file 1: Figure S1.**


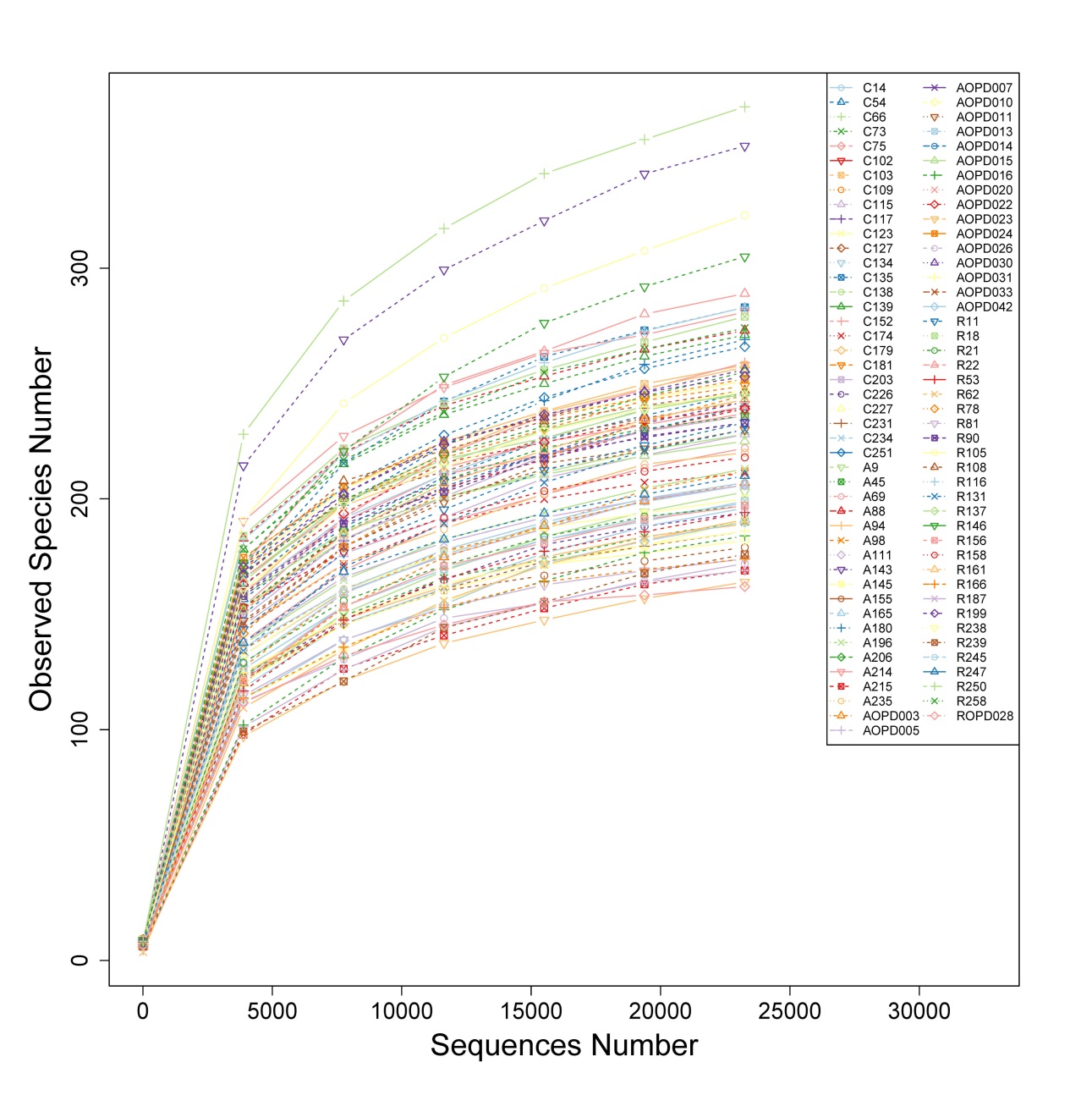


**Figure S1:** Rarefaction curves of operational taxonomic units (OTUs) diversity for each sample. Each of the stool samples contains 23,254 sequences to obtain equal sampling depth.
