# Supplementary material for: Gut microbial dysbiosis is associated with allergen-specific IgE responses in young children with airway allergies
Source: World Allergy Organ J. 2019 Mar 25;12(3):100021. doi: 10.1016/j.waojou.2019.100021 (PMC6439417; doi:10.1016/j.waojou.2019.100021)
Supplement: Multimedia component 2 [file mmc2.docx]

**Additional file 2: Figure S2.**


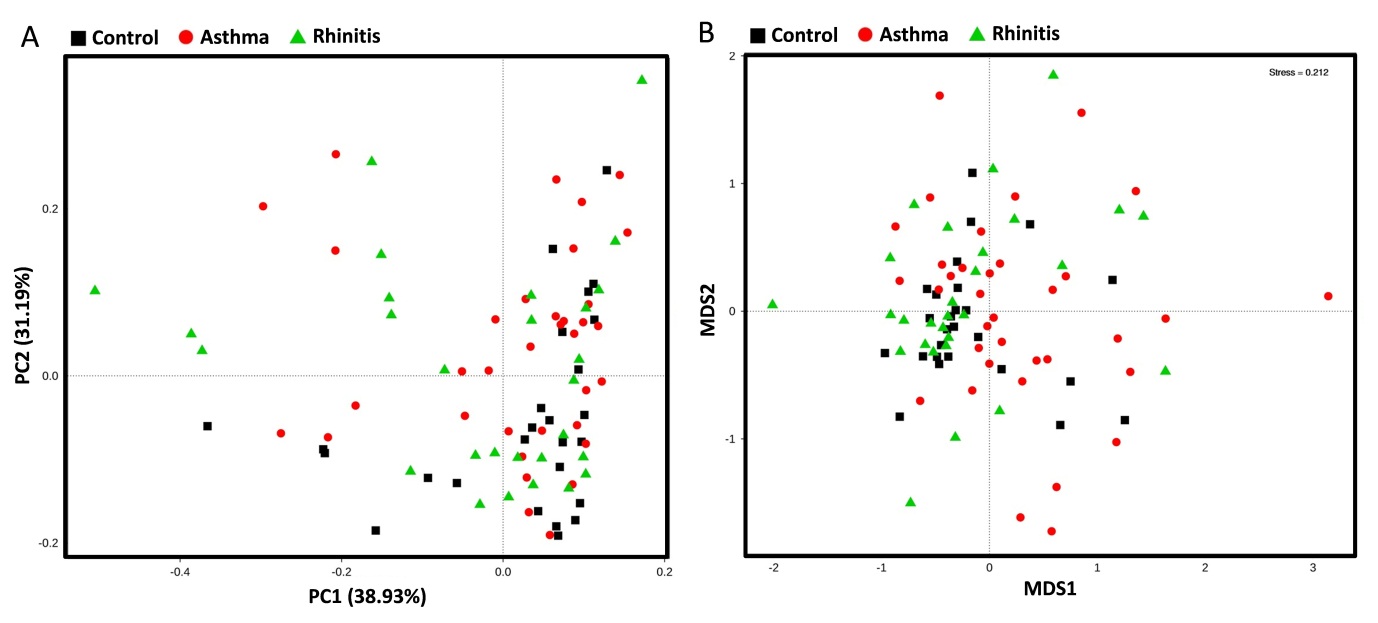


**Figure S2:** Beta diversity statistics using the Principal Coordinate Analysis (PCoA) and non-metric multidimensional scaling (NMDS). (A) PCoA plot of weighted Unifrac distance matrix showing patterns of beta diversity in bacterial communities. (B) Bray-Curtis based NMDS plot showing similarities in bacterial community composition between samples.
